# Identifying Influential Theories in Human–Computer Interaction Within Health Informatics: A Systematic Review

**DOI:** 10.1002/hsr2.71074

**Published:** 2025-07-21

**Authors:** Niloofar Mohammadzadeh, Fatemeh Lotfi, Hamed Samadpour

**Affiliations:** ^1^ Department of Health Information Management, School of Allied Medical Sciences Tehran University of Medical Sciences Tehran Iran

**Keywords:** health informatics, health information technology, human–computer interaction, theory, user‐centered design

## Abstract

**Background and Aims:**

Digital transformation in healthcare has driven the adoption of diverse health information systems, including electronic health records, mobile health (mHealth) applications, and clinical decision support tools. However, optimal interaction between users and these systems remains a challenge. The field of Human–Computer Interaction (HCI) offers theoretical frameworks to guide the design and evaluation of these technologies, enhancing usability and adoption by health professionals and patients. This systematic review aims to identify and synthesize the most frequently applied HCI theories in health informatics and examine their relevance across digital health technologies.

**Methods:**

A systematic review was conducted following PRISMA 2020 guidelines. Three databases—PubMed, Web of Science, and Google Scholar—were searched with no date restriction. Studies were included if they applied a defined HCI theory to the context of health information technologies and were published in English. Review articles, conceptual papers, and studies without a clear theoretical framework were excluded. A total of 67 eligible studies were included in the final synthesis.

**Results:**

The use of HCI theories in health information technology has increased notably since 2015, with a sharp rise observed after 2020. The most frequently used frameworks included the Unified Theory of Acceptance and Use of Technology (UTAUT), Technology Acceptance Model (TAM), and Social Cognitive Theory (SCT). mHealth applications accounted for most theory applications (37%), followed by hospital information systems and web‐based tools. The geographic distribution revealed that the United States, the United Kingdom, and Australia produced the highest number of theory‐driven studies.

**Conclusions:**

HCI theories are vital in improving the design, usability, and implementation of health information systems. This review underscores the importance of integrating user‐centered theoretical frameworks in system development and calls for broader geographic and contextual diversity in future theory‐based research.

## 1 Introduction

The rapid digitalization of the healthcare industry has led to the widespread adoption of various health information technologies (HIT), including electronic health records (EHRs), mobile health (mHealth), telemedicine platforms, wearable devices, and clinical decision support systems (CDSS). These systems are increasingly integral to clinical workflows, diagnostic processes, and patient engagement [1]. These technologies have boosted the efficacy of treatment approaches and improved diagnostic accuracy, thereby enhancing patient outcomes [2]. Despite these advances, many healthcare professionals and patients face challenges in effectively interacting with digital health tools. A recent study by HIMSS [3] revealed that doctors and nurses often struggle with managing tasks, integrating data, and interpreting the information displayed on these systems. It has been shown that usability issues, cognitive overload, and lack of alignment with clinical tasks can hinder adoption and limit the impact of these technologies.

Human–Computer Interaction (HCI) is an interdisciplinary field that draws from computer science, psychology, design, and cognitive science to examine and improve how humans interact with digital systems. In healthcare, HCI offers theoretical frameworks that guide user‐centered systems' design, evaluation, and implementation, aiming to enhance usability, performance, and satisfaction [4]. Originating in the early 1980s and focusing on enhancing user performance through interface design, HCI has since evolved into a robust domain that integrates cognitive science research and real‐world applications to validate theoretical models [5]. In healthcare, HCI research has progressed alongside general methodological advancements. Starting with cognitive evaluations of EHRs in the mid‐1990s [6], HCI in healthcare has expanded to encompass distributed health information systems [7]. It has also examined the unintended sociotechnical impacts of computerized provider order entry systems [8]. To reflect the diversity of systems examined, ranging from mobile apps and telehealth to hospital‐based informatics, we use the term “HCI” alongside broader expressions such as “health technology interactions” and “digital health interfaces.”

Theory plays a crucial role in HCI research and practice. Bederson and Shneiderman [9] identified five fundamental categories of HCI theories: generative, explanatory, predictive, prescriptive, and descriptive. Descriptive theories provide foundational concepts and methods, while explanatory theories clarify underlying processes. Predictive theories offer insights into user performance, while prescriptive theories guide design practices. Generative theories, on the other hand, propose new design ideas and interactive paradigms. In HIT, HCI Theories are critical for designing systems that address the specific needs of healthcare providers and patients [10].

Given the growing complexity of HIT systems and the demand for intuitive, efficient, and safe interfaces, there has been an increasing focus on theory‐based system design. Applying HCI theories helps researchers and developers predict, evaluate, and optimize user behavior and technology interaction. In recent years, the use of formal theoretical models, such as the Unified Theory of Acceptance and Use of Technology (UTAUT), Technology Acceptance Model (TAM), and Social Cognitive Theory (SCT), has grown, becoming essential in guiding system evaluation and implementation.

This systematic review identifies and synthesizes the most frequently applied HCI theories in health informatics research, focusing on their application across different HIT systems. The review also examines how theories are used over time and across geographies, offering a broader perspective on how these frameworks support technology adoption and user engagement in healthcare.

To identify relevant literature, we searched three major databases: PubMed, Web of Science, and Google Scholar. These were chosen for their broad coverage of biomedical and interdisciplinary literature. While we acknowledge other databases like ACM Digital Library, AIS eLibrary, and IEEE Xplore, we prioritized databases specific to health informatics to maintain domain specificity. These choices allowed us to concentrate on studies directly within healthcare delivery and practice, rather than general computing or information systems environments.

This review highlights key findings, including the growing application of UTAUT, TAM, and SCT in various health informatics systems, particularly in mHealth. It contributes to the field by:

1. Mapping the landscape of HCI theory use in HIT.
2. Highlighting dominant frameworks and their practical applications.
3. Identifying research gaps and future directions, emphasizing the need for more context‐aware and geographically diverse research.

## 2 Methods

**Figure 1:**
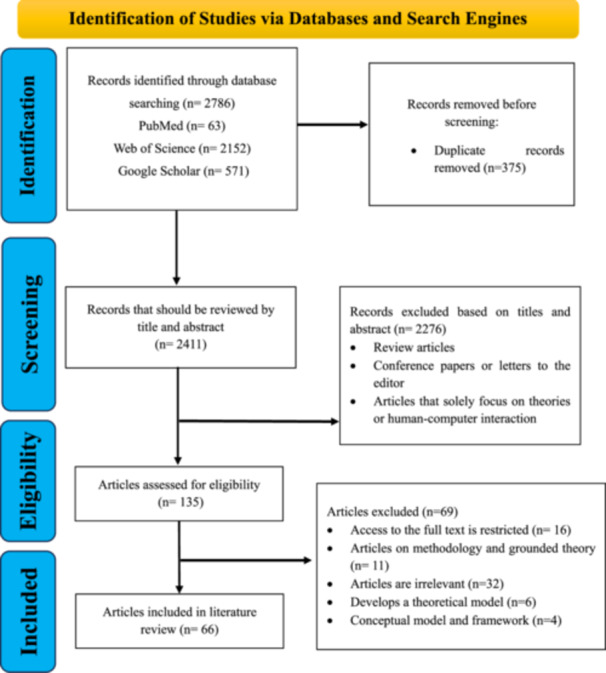
PRISMA flow diagram of literature search and study selection process.

This study was conducted as a systematic review following the Preferred Reporting Items for Systematic Reviews and Meta‐Analyses (PRISMA 2020) guidelines to ensure rigor, transparency, and reproducibility. A PRISMA flow diagram (Figure 1) illustrated the screening and selection process, and a completed PRISMA checklist guided reporting [11].

### 2.1 Literature Search

A comprehensive literature search was conducted across three major databases: PubMed, Web of Science, and Google Scholar. These databases were selected based on their broad and complementary coverage of biomedical, interdisciplinary, and gray literature:

- *PubMed* indexes clinical and biomedical informatics literature;
- *Web of Science* captures cross‐disciplinary studies, including behavioral and social dimensions of health technologies; and
- *Google Scholar* was used to maximize the retrieval of potentially relevant.

The search strategy included combinations of keywords and controlled vocabulary terms (e.g., MeSH, thesaurus terms) related to HCI, health informatics systems, and theoretical frameworks. The publication date was not restricted. Table 1 presents the full search syntax used for each database.

**Table 1:** Search strategies used across databases.

Database name	Search query	Result
Pubmed	(Human Computer Interaction[Title/Abstract] OR HCI[Title/Abstract] OR User Experience[Title/Abstract] OR UX[Title/Abstract] OR User Satisfaction[Title/Abstract] OR Usability[Title/Abstract] OR User Empowerment[Title/Abstract] OR User Engagement[Title/Abstract] OR User Interaction[Title/Abstract] OR User Centered Design[Title/Abstract] OR UCD[Title/Abstract] OR Interaction Design[Title/Abstract] OR Interface Design[Title/Abstract] OR Human Factors[Title/Abstract] OR System Feedback[Title/Abstract] OR User Interface Guidelines[Title/Abstract] OR user attitude[Title/Abstract] OR Prototyping[Title/Abstract]) AND (Health Informatics[Title/Abstract] OR medical informatics[Title/Abstract] OR eHealth[Title/Abstract] OR electronic health[Title/Abstract] OR mHealth[Title/Abstract] OR mobile health[Title/Abstract] OR telemedicine[Title/Abstract] OR healthcare applications[Title/Abstract] OR healthcare apps[Title/Abstract] OR medical software[Title/Abstract] OR electronic health information system[Title/Abstract] OR hospital information system[Title/Abstract] OR hospital communication system[Title/Abstract] OR clinical decision support system[Title/Abstract]) AND (Health informatics theory [Title/Abstract] OR Effective Theories[Title/Abstract] OR Cognitive Models[Title/Abstract] OR Technology Adoption[Title/Abstract] OR Health Technology Evaluation[Title/Abstract] OR Social theory[Title/Abstract] OR behavior change theory[Title/Abstract] OR behavioral theory[Title/Abstract] OR theory‐based design[Title/Abstract] OR management theory[Title/Abstract] OR learning theory[Title/Abstract] OR sociotechnical theory[Title/Abstract] OR activity theory[Title/Abstract] OR action theory[Title/Abstract])	63
Web of Science	AB = Abstracts(AB = (Human Computer Interaction) OR AB = (HCI) OR AB = (User Experience) OR AB = (UX) OR AB = (User Satisfaction) OR AB = (Usability) OR AB = (User Empowerment) OR AB = (User Engagement) OR AB = (User Interaction) OR AB = (User Centered Design) OR AB = (UCD) OR AB = (Interaction Design) OR AB = (Interface Design) OR AB = (Human Factors) OR AB = (System Feedback) OR AB = (User Interface Guidelines) OR AB = (user attitude) OR AB = (Prototyping)) AND (AB = (Health Informatics) OR AB = (medical informatics) OR AB = (eHealth) OR AB = (electronic health) OR AB = (mHealth) OR AB = (mobile health) OR AB = (telemedicine) OR AB = (healthcare applications) OR AB = (healthcare apps) OR AB = (medical software) OR AB = (electronic health information system) OR AB = (hospital information system) OR AB = (hospital communication system) OR AB = (clinical decision support system)) AND (AB = (Health informatics theory) OR AB = (Effective Theories) OR AB = (Cognitive Models) OR AB = (Technology Adoption) OR AB = (Health Technology Evaluation) OR AB = (Social theory) OR AB;= (behavior change theory) OR AB = (behavioral theory) OR AB = (theory‐based design) OR AB = (management theory) OR AB = (learning theory) OR AB = (sociotechnical theory) OR AB = (activity theory) OR AB = (action theory))OR TI = Title(TI = (Human Computer Interaction) OR TI = (HCI) OR TI = (User Experience) OR TI = (UX) OR TI = (User Satisfaction) OR TI = (Usability) OR TI = (User Empowerment) OR TI = (User Engagement) OR TI = (User Interaction) OR TI = (User Centered Design) OR TI = (UCD) OR TI = (Interaction Design) OR TI = (Interface Design) OR TI = (Human Factors) OR TI = (System Feedback) OR TI = (User Interface Guidelines) OR TI = (user attitude) OR TI = (Prototyping)) AND (TI = (Health Informatics) OR TI = (medical informatics) OR TI = (eHealth) OR TI = (electronic health) OR TI = (mHealth) OR TI = (mobile health) OR TI = (telemedicine) OR TI = (healthcare applications) OR TI = (healthcare apps) OR TI = (medical software) OR TI = (electronic health information system) OR TI = (hospital information system) OR TI = (hospital communication system) OR TI = (clinical decision support system)) AND (TI = (Health informatics theory) OR TI = (Effective Theories) OR TI = (Cognitive Models) OR TI = (Technology Adoption) OR TI = (Health Technology Evaluation) OR TI = (Social theory) OR TI = (behavior change theory) OR TI = (behavioral theory) OR TI = (theory‐based design) OR TI = (management theory) OR TI = (learning theory) OR TI = (sociotechnical theory) OR TI = (activity theory) OR TI = (action theory))	2152
Google Scholar	“theory” AND “health informatics” AND “human–computer interactions”	571
Total		2786

### 2.2 Inclusion and Exclusion Criteria

Eligibility criteria were defined a priori to ensure the relevance, quality, and theoretical focus of included studies. Articles were included if they:

- were published in English with full‐text access; and
- were empirical studies that applied a named HCI theoretical framework related to digital health systems. Eligible studies included theory‐driven system design, evaluation, testing, behavioral analysis, or implementation processes involving technologies such as EHRs, CDSS, mHealth apps, or telehealth platforms. Examples include studies that applied the TAM to assess clinician adoption of EHRs or used SCT to guide the development of mHealth applications.

Studies were excluded if they:

- were review articles, editorials, letters, or conference abstracts;
- focused solely on methodology or did not apply a defined theory; and
- addressed general HCI without reference to health or healthcare settings.

### 2.3 Study Selection and Data Extraction Phase

After completing the identification and screening process, the study team reviewed and discussed the selected publications in two‐ to three‐person sessions, following PRISMA 2020 guidelines to ensure transparency and reproducibility. The initial database search retrieved 2786 records, of which 375 duplicates were removed. Of the remaining 2411 records, 2276 were excluded based on title and abstract screening. The eligibility of 135 full‐text publications was then assessed, with 69 excluded for being review papers, lacking full‐text access, or containing unrelated content. Ultimately, 66 publications were included in the PRISMA process. Articles requiring further analysis or clarification were referred to the third author. The PRISMA chart (Figure 1) illustrates the exclusion process at each stage, including criteria such as review articles, conference papers, letters to the editor, and publications without a theoretical focus or relevance to HCI. Although our initial search strategy was designed to be comprehensive, one eligible study by Or et al. [12] was identified during peer review and subsequently assessed using the same inclusion criteria. As it met all predefined requirements, it was incorporated into the final synthesis, although it is not reflected in the PRISMA flow counts. With this addition, the total number of studies included in the final synthesis has increased to 67.

## 3 Results

This study's findings highlight the critical role of HCI theories in health informatics. The results are categorized by year of publication, geographic distribution, and health informatics systems. These findings provide a comprehensive perspective on how HCI theories shape the field across different contexts. This growing body of theory‐driven research reflects an increasing emphasis on improving system usability, enhancing technology adoption, and aligning system design with users' cognitive and contextual needs.

### 3.1 Usage of HCI Theories Over Time

**Figure 2:**
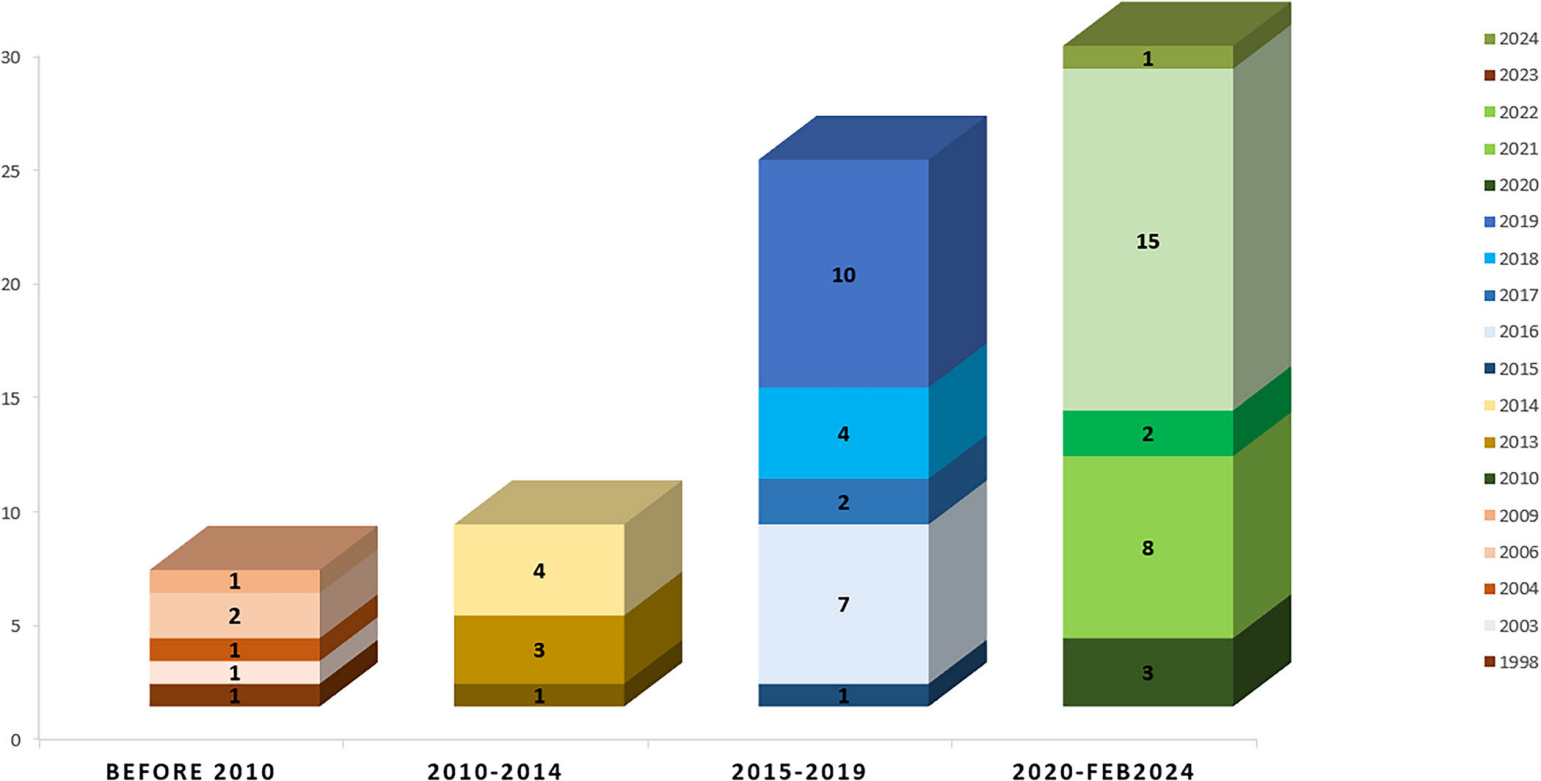
Distribution of the number of research papers by year.

Figure 2 presents the final articles, categorized by publication year. It is a bar chart depicting the number of research studies conducted over four distinct periods: before 2010, 2010–2014, 2015–2019, and 2020–February 2024. This chart provides insight into the temporal trends in adopting and applying theories in health systems research, revealing a significant increase in studies from 2015 onwards, with the most substantial growth observed after 2020. This trend may be attributed to the increasing complexity of health information systems, the broader integration of user‐centered design practices, and a stronger demand for evidence‐based evaluation of digital health interventions.

Complementing this visual representation, Table 2 offers a comprehensive overview of the application of HCI theories across these four distinct periods, detailing the frequency of each theory's usage during these intervals. The data highlight a clear upward trend in adopting theoretical frameworks in health informatics research. For instance, the UTAUT has demonstrated a marked increase in usage since 2015, with a significant rise after 2020, indicating the growing focus on technology acceptance in recent years. Similarly, the SCT and the Theory of Planned Behavior (TPB) have experienced a notable rise in application, further demonstrating the expanding integration of these theories in health‐related studies. These patterns reflect broader shifts in the field, where researchers are increasingly turning to well‐established theoretical models to evaluate user interaction, predict behavior, and design more adaptable and user‐centered systems. Such frameworks are instrumental in managing the complexity of digital health environments and ensuring that interventions are both effective and aligned with the practical needs of healthcare professionals and patients.

**Table 2:** Frequency of HCI theories used in the reviewed studies across publication year ranges.

HCI Theory/Number of studies per publication year range	Before 2010	2010–2014	2015–2019	2020–now	Total
Activity Theory [13, 14, 15]	—	—	1	2	3
Actor‐Network Theory [16, 17]	—	1	1	—	2
Behavior Change Theory [18, 19, 20, 21, 22]	—	2	1	2	5
Behavioral Science Theory [23, 24, 25]	—	—	1	2	3
Domain‐Theory [26]	1	—	—	—	1
Flow Theory [27]	—	—	—	1	1
Game Theory [28]	—	—	—	1	1
Innovation Theory [29]	—	—	1	—	1
Lewin's Force Field Theory of Change [30]	1	—	—	—	1
Normalization Process Theory (NPT) [31, 32, 33, 34, 35]	—	1	2	2	5
Resource Dependence Theory (RDT) [36]	—	—	1	—	1
Self‐Regulation Theory (SRT) [37]	—	—	1	—	1
Self‐Determination Theory (SDT) [38, 39]	—	—	1	1	2
Social Cognitive Theory (SCT) [40, 41, 42, 43]	—	—	1	3	4
Social‐Cognition Models of Health Behavior—Willingness [44]	1	—	—	—	1
Technology Acceptance Model (TAM) [45, 46, 47, 48]	1	1	1	1	4
Post‐Acceptance Model (PAM) and the Technology Acceptance Model (TAM) [49]	—	—	1	—	1
Technology Readiness Model and Perceived Value Theory [50]	—	—	—	1	1
Cognitive Walk‐Through (CW) [50]	1	—	—	—	1
Institutional Theory [51]	—	—	1	—	1
The Adaptive Control of Thought‐Rational (ACT‐R) Theory of Cognition [52]	—	—	1	—	1
The Situation‐Specific Theory of Heart Failure Self‐Care [53]	—	—	1	—	1
The Sociotechnical Systems Theory [54]	—	1	—	—	1
The Theory of Triangulation [55]	1	—	—	—	1
The Wellness Motivation Theory (WMT) [56]	—	1	—	—	1
Theory of Change (ToC) [57]	—	—	—	1	1
Theory of Planned Behavior (TPB) [58, 59, 60, 61]	—	—	1	3	4
Theory of Reasoned Action [62]	—	—	1	—	1
Unified Theory of Acceptance and Use of Technology (UTAUT) [12, 63, 64, 65, 66, 67, 68, 69, 70, 71, 72, 73, 74, 75, 76]	—	1	6	8	15
Unified Theory of Acceptance and Use of Technology 2 (UTAUT2) [77]	—	—	—	1	1
Total	6	8	24	29	67

*Note:* Only the primary theory emphasized in each study is listed.

### 3.2 Geographic Distribution of Research Papers

**Figure 3:**
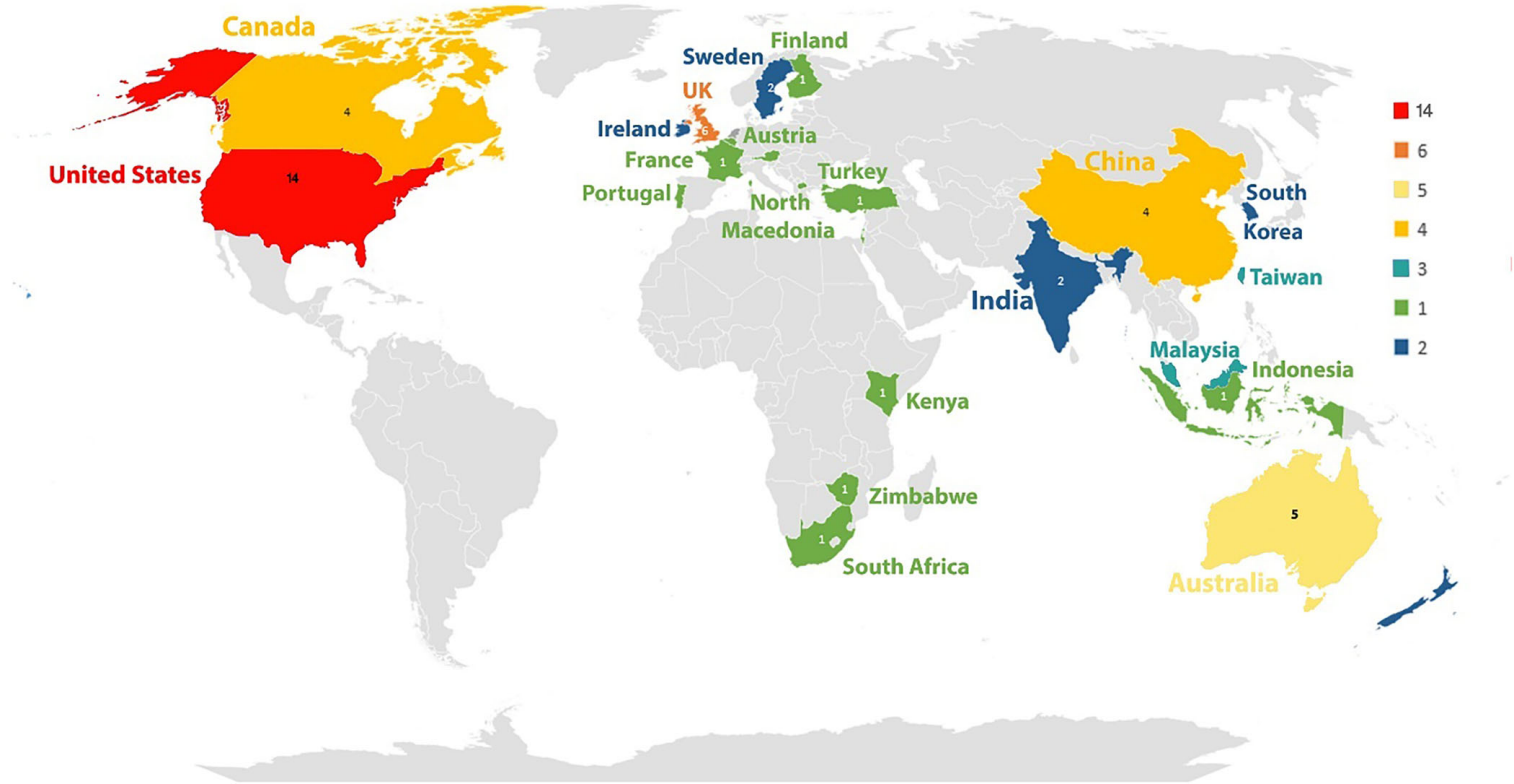
Geographic distribution of research papers on HCI theories in health informatics (1998–2024).

Different regional contributions are shown by the global landscape of research on HCI theories in health informatics, highlighting the proactive approach of certain countries in developing this subject. Figure 3 demonstrates the regional distribution of research papers employing HCI ideas within health informatics. The United States ranks first with 14 studies, followed by the United Kingdom with 6, and Australia with 5. All these indicate these nations' significant contributions to advancing HCI theory applications in health systems. This trend may be linked to advanced digital health infrastructures, better institutional support for research, and higher levels of technology adoption readiness in these countries. The strong representation of high‐income regions suggests that applying HCI theories often depends on contextual factors such as policy frameworks, funding availability, and digital literacy. This pattern also highlights the need for more inclusive global research efforts, particularly in underrepresented regions, to ensure broader applicability and cultural relevance of theory‐driven approaches.

### 3.3 Distribution of HCI Theories by Health Informatics Systems

**Figure 4:**
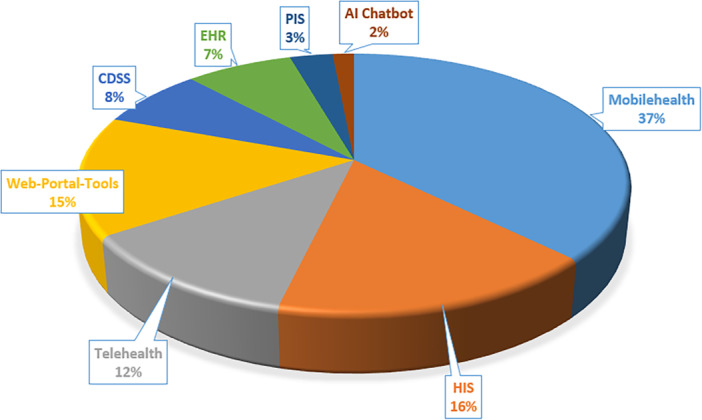
Distribution of HCI theories by health informatics systems.

The distribution of health informatics systems highlights the application of various HCI theories presented in Figure 4. This analysis includes systems such as AI chatbots, CDSS, EHRs, Hospital Information Systems (HIS), mHealth, Pharmacy Information Systems (PIS), Telehealth, and Web‐Based Portals and Tools. mHealth systems account for the most significant proportion of HCI theory applications, representing 37% of all instances. This is followed by HIS (16%), Web‐Based Portals and Tools (15%), and Telehealth (12%). Other systems, including CDSS, EHR, PIS, and AI chatbots, exhibit more limited usage, with lower representation ranging from 8% to 2%.

The prominence of mHealth may reflect the high degree of user interaction and personalization required in mobile applications, making them a natural focus for theory‐driven evaluation. Conversely, systems such as CDSS or PIS may involve more backend automation and less direct user input, which could explain their lower frequency of theoretical application. This distribution suggests that theory use is not uniform across systems and is likely influenced by the nature of user engagement, system complexity, and the level of interaction required. Recognizing these distinctions can help guide the selection of appropriate theoretical frameworks based on system type and user context.

Table 3 provides a comprehensive breakdown of the application of various HCI theories across different health informatics systems, offering insights into the diversity and prevalence of theoretical frameworks within each system type. The UTAUT is the most widely utilized framework, with 15 instances predominantly applied in mHealth systems. This reflects its effectiveness in addressing user acceptance and engagement in mobile‐based healthcare applications. Similarly, behavior change theory, SCT, and self‐determination theory (SDT) have frequently been employed in mHealth contexts. Other frameworks, including Activity Theory, Normalization Process Theory (NPT), SCT, TAM, TPB, and UTAUT, have shown versatility across multiple systems.

**Table 3:** Application of HCI theories across different health informatics systems.

Theory/System	AI Chatbot	CDSS	HER	HIS	Mobile Health	PIS	Telehealth	Web‐Portal‐Tools	Total
Activity Theory			✓		✓	✓			3
Actor‐Network Theory				✓✓					2
Behavior Change Theory					✓✓✓✓✓				5
Behavioral Science Theory		✓			✓				2
Domain Theory							✓	✓	2
Flow Theory							✓		1
Game Theory		✓							1
Innovation Theory				✓					1
Lewin's Force Field Theory of Change				✓					1
Normalization Process Theory (NPT)			✓✓✓		✓		✓		5
Resource Dependence Theory (RDT)				✓					1
Self‐Regulation Theory (SRT)								✓	1
Self‐Determination Theory (SDT)					✓✓				2
Social Cognitive Theory (SCT)					✓✓		✓	✓	4
Social Cognition Models of Health Behavior‐Willingness							✓		1
Technology Acceptance Model (TAM)				✓✓	✓			✓	4
Post‐Acceptance Model (PAM) and the Technology Acceptance Model (TAM)					✓				1
Technology Readiness Model and Perceived Value Theory							✓		1
Cognitive Walk‐Through (CW)				✓					1
Institutional Theory				✓					1
The Adaptive Control of Thought‐Rational (ACT‐R) Theory of Cognition					✓				1
The Situation‐Specific Theory of Heart Failure Self‐Care					✓				1
The Sociotechnical Systems Theory		✓							1
The Theory of Triangulation				✓					1
The Wellness Motivation Theory (WMT)					✓				1
Theory of Change (ToC)					✓				1
Theory of Planned Behavior (TPB)					✓		✓	✓✓	4
Theory of Reasoned Action					✓				1
Unified Theory of Acceptance and Use of Technology (UTAUT)	✓	✓✓	✓	✓	✓✓✓✓✓	✓	✓	✓✓✓	15
Unified Theory of Acceptance and Use of Technology 2 (UTAUT2)								✓	1
Total	1	5	5	11	25	2	8	10	67

*Note:* Only the primary theory emphasized in each study is listed.

The repeated use of these frameworks across different health IT environments suggests conceptual flexibility and practical adaptability to diverse healthcare settings and interaction models. For example, theories such as TAM, TPB, and UTAUT are often chosen for their robust explanatory power in user adoption, making them suitable for individual‐centered tools (e.g., mHealth apps) and enterprise‐level platforms (e.g., HIS, EHRs).

The frequent application of UTAUT and TAM in mHealth and web‐based tools may be due to their strong focus on acceptance constructs such as perceived usefulness and ease of use, which are particularly critical in voluntary‐use systems. In contrast, frameworks like NPT and Activity Theory are more often applied in complex systems such as HIS and EHRs, where the emphasis shifts toward organizational integration, workflow alignment, and sociotechnical dynamics.

To enhance conceptual clarity, the frameworks identified in this review can be broadly categorized into three functional groups: (1) acceptance models (e.g., TAM, UTAUT, TPB), which focus on users' intention to adopt and continue using digital health tools; (2) behavior change frameworks (e.g., SCT, SDT, Transtheoretical Model), which emphasize motivation, intention formation, and sustained engagement; and (3) sociotechnical and cognitive theories (e.g., Activity Theory, NPT), which analyze how technology becomes embedded in collective practices and organizational routines. This classification clarifies the theoretical landscape and reveals how different frameworks align with varying types of systems, user goals, and contexts of use.

### 3.4 Users' Attitudes and Perceptions

Another aspect with an essential role in accepting new technologies is user attitudes. We can gain insights into the efficacy of these theories and frameworks from a user perspective, which is crucial for understanding their practical application and impact on health informatics. Table 4 summarizes various studies on user acceptance and perceptions of HIT revealing several vital factors influencing the adoption and continued use.

**Table 4:** Summary of critical information and user perceptions from the selected articles.

Authors	Year of publication	Country	Study objective	Theories used	Type of electronic system	Key findings	User perception	HCI application	Recommendations
Hyun et al. [45]	2009	USA	Exploring nurses' perceptions of electronic documentation	Technology Acceptance Model (TAM), Task Technology Fit (TTF)	Health Information System (HIS)	High usability, useful, well‐matched to tasks	Easy to use, potentially useful	User‐centered design, theory‐based methods	Improving interface design based on user feedback
Bouamrane and Mair [31]	2013	UK	Assessing GPs' perspectives on EMR systems	Normalization Process Theory (NPT)	Electronic Health Record (EHR)	Mixed satisfaction, usability issues, and training needed	Essential but needs improvement	User‐centered design, iterative improvements	Improving usability, providing training
Yuan et al. [63]	2015	USA	Examining predictors of continued use of health and fitness apps	Extended Unified Theory of Acceptance and Use of Technology (UTAUT2)	Mobile Health	Significant predictors: performance expectancy, hedonic motivation, price value, and habit	High importance on performance, fun, price, and habit	User‐centered design, gamification, and pricing strategies	Focusing on performance, fun, value, and habit formation
English et al. [66]	2016	USA	Evaluating acceptance of CDSS among clinical pharmacists	Unified Theory of Acceptance and Use of Technology (UTAUT)	Clinical Decision Support System (CDSS)	Satisfaction influences use; facilitating conditions are important	Satisfaction reduces workarounds	User satisfaction, system facilitation	Enhancing organizational support, improving user satisfaction
Nguyen et al. [17]	2017	Australia	Investigating nurses' reactions to electronic documentation	Actor‐Network Theory (ANT)	Health Information System (HIS)	Usability issues; improved information access	Frustration with system crashes; benefits in later stages	User‐centered design, iterative improvements	Enhancing auto‐save features, improving training
Hägglund et al. [53]	2019	Sweden	Testing heart failure self‐care theory using mHealth	Situation‐Specific Theory of Heart Failure Self‐care	Mobile Health	Feasible, supports self‐care, fosters independence	Positive, increased adherence, skill development	User‐centered design, iterative feedback	Enhancing technical support, integrating with healthcare professionals
Nie et al. [48]	2023	Canada	Generating transferrable lessons for future designers of health information technology tools that facilitate team communication and collaboration.	Technology Acceptance Model (TAM)	Web‐Portal‐Tools	‐Facilitating learning and use through intuitive design ‐Enhancing information organization to create a comprehensive clinical picture	‐Generally positive, found the tool easy to use and adopt. ‐Frustration with specific functionalities not being discovered.	User‐centered design, iterative improvements, and addressing both technological and nontechnological factors.	Enhancing user training to ensure the discovery of all features.
Richardson et al. [25]	2023	USA	Employing a user‐centered design process to develop a CDS tool	Behavioral Science Theory	Clinical Decision Support System (CDSS)	‐Facilitating learning and use through intuitive design ‐Enhancing information organization to create a comprehensive clinical picture	Clinicians highlighted several significant psychological and behavioral barriers to CDS use and mixed feelings about the coexistence of paper and electronic systems.	User‐centered design, iterative improvements, addressing both technological and nontechnology	Focusing on mixed methods for barrier identification. Addressing psychological barriers to enhance CDS adoption.

Additionally, usability and adequate user training are essential elements that facilitate improved user experience and increase system satisfaction [31, 48]. User‐centered design and iterative improvements have often led to enhanced efficiency and greater technology acceptance [17, 25, 45, 48, 53, 63, 66]. Performance expectations, user habits, and facilitating conditions have also been identified as significant factors in adopting these technologies [63]. Users are more likely to adopt systems perceived as functional, easy to use, and sufficiently supported by their organizations [45, 48].

Further, intuitive design and information organization that simplify system use play a crucial role in user acceptance [25, 48]. However, challenges such as the coexistence of paper‐based and electronic systems and the need for enhanced training to ensure full utilization of system features have also been highlighted [25]. These findings indicate that successful adoption of HIT depends on technical features and aligning system design with user expectations, behavioral tendencies, and organizational support structures. The interaction between user experience factors and theoretical constructs (such as performance expectancy, effort expectancy, and social influence) plays a central role in shaping real‐world outcomes.

A comprehensive examination of the existing literature indicates a notable increase in scholarly investigations concerning implementing HCI theories within healthcare frameworks, particularly following 2015, with an even more pronounced acceleration noted since 2020. Among the various implementations, mHealth systems have demonstrated the most significant prevalence, comprising 37% of studies grounded in HCI theory, followed by HIS (16%) and web portals (15%). Among the diverse theoretical frameworks utilized, the UTAUT has been the most extensively applied, particularly within mHealth systems and CDSS. Regionally, the predominant body of research has emerged from the United States, the United Kingdom, and Australia, suggesting a concentrated scholarly emphasis within these countries. This distribution may reflect differences in digital health maturity, investment in research infrastructure, and institutional capacity to adopt theory‐based evaluation frameworks.

Furthermore, the findings underscore that user‐centered design, usability, and comprehensive training have been critical in improving user satisfaction and the adoption rates of these systems. Nonetheless, usability challenges and inadequate training have also been acknowledged, particularly in more intricate systems. These insights reinforce the importance of applying HCI theories in context‐sensitive ways that are responsive to both system type and user realities.

## 4 Discussion

Theoretical frameworks identified in this review can be conceptually grouped into three categories: (1) technology acceptance models (e.g., UTAUT, TAM, TPB), which focus on users' intention to adopt digital health systems; (2) behavior change theories (e.g., SCT, SDT, Transtheoretical Model), which explain motivational and psychological aspects of long‐term engagement; and (3) sociotechnical and cognitive integration frameworks (e.g., Activity Theory, NPT), which analyze how digital tools are embedded into organizational routines and collaborative workflows. This categorization provides a more transparent lens for analyzing how different health informatics systems benefit from specific theoretical perspectives.

These dominant frameworks, such as UTAUT, TAM, SCT, and TPB, reflect broader themes in user‐centered design and behavior change. Their widespread adoption across health informatics research underscores a collective emphasis on core areas like usability, engagement, and system acceptance, which are central to ensuring the success of digital health technologies.

The findings of this study underscore the growing relevance of HCI theories in the design, implementation, and evaluation of health informatics systems, particularly as they relate to the increasing adoption of mHealth technologies. HCI theories have been significantly applied in various health informatics systems, with a notable focus on mHealth systems since 2015. The UTAUT has emerged as the most widely utilized framework, especially after 2020, highlighting the importance of technology acceptance by users in this field. UTAUT's emphasis on user perceptions of ease of use, performance expectancy, and social influence has proven especially relevant in understanding user engagement with mobile technologies. This growing application of UTAUT reflects the necessity for user‐centered approaches to promote technology adoption and enhance patient self‐management in mHealth systems. This trend suggests a shift toward frameworks that explain user behavior and inform proactive design decisions in technology development.

The increasing use of HCI theories in health informatics research can be attributed to several interrelated factors. Modern health information systems are becoming more complex, with integrated EHRs, AI‐assisted decision support, and mHealth technologies requiring structured frameworks for effective design and evaluation. Additionally, there is a growing emphasis on user‐centered design as digital tools must align with user needs and clinical workflows to maximize engagement and adoption. The rising complexity of systems, combined with the need for effective technology adoption, has driven the integration of theory‐based approaches into the development and evaluation of digital health tools. Moreover, the increasing demand for evidence‐based implementation in healthcare systems has made theoretical frameworks more relevant, as they provide a rigorous foundation for measuring user engagement, satisfaction, and system effectiveness.

In addition to UTAUT, SCT and the TPB have been widely applied in recent years, demonstrating the importance of understanding users' psychological and behavioral factors for motivating and sustaining long‐term interaction with health technologies. These theories have been utilized in various health systems, particularly chronic disease management and telehealth, contributing to improved user engagement and technology acceptance. Behavioral frameworks such as the Theory of Behavioral Change and SDT have also been extensively used in mHealth to induce long‐term behavioral changes, further aiding in designing systems that foster user motivation, self‐efficacy, and sustained engagement. Such frameworks enable systems design that moves beyond one‐time use and fosters meaningful behavior change over time.

Beyond the extensively discussed frameworks, several other conceptually rich models—such as NPT, Activity Theory, and the ACT‐R cognitive architecture—offer important perspectives that enrich our understanding of user interaction and system integration. NPT provides valuable insights into how health technologies are integrated into routine clinical workflows, emphasizing the importance of collective action and team dynamics in successful implementation. This makes it especially relevant to complex systems, such as EHRs and CDSS. Activity Theory, with its emphasis on mediated action and contextual contradictions, sheds light on how users adapt to systems in real‐world environments and how unintended consequences may emerge during implementation. Meanwhile, ACT‐R offers a computational model of human cognition that can predict user behavior and inform interface design, particularly in high‐stakes or cognitively demanding settings.

These frameworks, although not as prominently featured in prior reviews, provide powerful analytical tools that complement dominant acceptance‐based models. Their inclusion supports a more comprehensive and theoretically diverse understanding of system use, adaptation, and cognitive demands in health informatics. Broadening the conceptual lens in this way helps reduce overreliance on a narrow subset of models. It also promotes a more inclusive and interdisciplinary approach to theory selection in HCI research.

The geographic distribution of studies in this review highlights regional disparities in digital health infrastructure, funding, and research support. While countries like the United States, the United Kingdom, and Australia lead in applying HCI theories in health informatics due to their advanced infrastructures, regions with less developed systems face barriers such as limited funding and resources, resulting in lower research output. Our intention was not to merely quantify publications by country but to explore how contextual differences across regions may shape the use and relevance of HCI theories in HIT. In countries with more advanced infrastructures, there is typically greater institutional support for research and organizational readiness for technology adoption. These favorable conditions increase both the necessity and feasibility of applying theoretical frameworks to guide system design, evaluation, and user engagement strategies. By contrast, regions with limited digital health integration often encounter fewer opportunities or incentives to implement theory‐driven assessments, leading to their underrepresentation in the literature.

This study concentration highlights the need for greater participation from underrepresented regions. Expanding efforts in these areas ensures health informatics theories remain relevant across diverse contexts and encourages the adaptation of HCI theories to meet the needs of low‐ and middle‐income countries. Addressing these disparities will make technological innovations more inclusive and promote a globally applicable approach to health informatics.

In addition to the empirical frameworks included in our review, we acknowledge the conceptual relevance of models from the fields of human factors, usability inspection, and implementation science, such as the frameworks discussed by Karsh et al. [78]. Emphasize ergonomics and the role of cognitive workload in system use; Or et al. [79, 80] focus on usability inspection methods and the evaluation of health IT from a human‐centered perspective; and Sittig and Singh [81] propose a sociotechnical model for understanding and mitigating unintended consequences of HIT implementation. While these sources were not part of our final data set due to indexing and format constraints, they offer valuable theoretical insight that complements the scope of our analysis.

From a user‐specific perspective, the study reveals the necessity of accounting for the diverse user groups interacting with health informatics systems. Healthcare providers prioritize system efficiency, ease of data entry, and rapid access to patient information, while patients, particularly in mHealth settings, value usability, personalization, and sustained engagement. Designing systems with varying levels of digital literacy in mind is essential for encouraging widespread adoption and ensuring long‐term usage. Future system designs should consider not only user roles but also levels of digital readiness and preferred modes of interaction.

Our findings align with prior work, such as the review by Heinsch et al. [82], which explored the application of theoretical frameworks in behavior change interventions within eHealth. While that study focused on health promotion and specific behavioral theories, our review covers a broader range of digital health systems, including EHRs, CDSS, HIS, mHealth, and telehealth. It examines the application of theories not only in system design but also in adoption, evaluation, and clinical integration. Additionally, our analysis provides a comparative overview of how theories have been applied across different periods and geographic regions.

Building on these insights, our analysis highlights several key directions for advancing health informatics:

1. *Deeper theoretical integration:* There is a critical need to bridge user‐centered design, behavioral science, and sociotechnical perspectives. This integration should move beyond isolated models to more holistic approaches that capture the full complexity of health informatics ecosystems.
2. *System‐specific theory application:* The application of theory must be tailored to specific systems, such as *mHealth*, *EHRs*, and *telehealth*, to maximize impact on user engagement and health outcomes.
3. *Addressing geographic disparities:* Tackling geographic disparities and underrepresented populations is crucial for achieving global inclusivity in health informatics research. Theory‐driven research must consider *cultural, infrastructural, and socioeconomic contexts* that affect the adoption and implementation of digital health technologies.
4. *Translating theory into actionable strategies:* Future research should focus on converting theoretical insights into practical strategies that improve health outcomes, reduce disparities, and foster sustainable implementation, ensuring that digital innovations meet the diverse needs of healthcare systems worldwide.

## 5 Limitations and Future Research

While this study provided valuable insights into the theoretical landscape of HCI in health informatics, it had limitations. First, the review was limited to studies published in English, which may have excluded relevant work from non‐English‐speaking regions.

Another limitation relates to our database selection strategy. We deliberately focused on PubMed, Web of Science, and Google Scholar to ensure that the review captured studies grounded in health informatics and biomedical research. While this approach helped maintain relevance to healthcare contexts, it may have excluded important interdisciplinary contributions. Specifically, databases such as ACM Digital Library, AIS eLibrary, and IEEE Xplore—where many foundational HCI frameworks, including cognitive models like UTAUT, were originally published—were not included in our selected database set and thus were excluded from the search process. As a result, specific theoretical perspectives from computing and information systems may be underrepresented.

Future research should consider broadening the scope of database coverage to include interdisciplinary and technical sources, as well as literature in languages other than English. Moreover, since most of the included studies originated from high‐income countries, there is a need for greater global representation in future research on HCI theory application.

Another limitation is that we recognize that specific theoretically relevant sources—including conceptual works, review articles, and book chapters—were excluded based on our predefined inclusion criteria, which prioritized empirical studies applying named HCI frameworks to real‐world systems. While such sources were not part of the systematic data set, their conceptual insights were reviewed and integrated into the discussion to complement our thematic analysis.

In addition, while this study focused on applying theories in specific health informatics systems, more empirical studies are needed to examine the effectiveness of these theories in practice. Longitudinal studies that track the long‐term impact of these theoretical applications on user satisfaction, health outcomes, and system efficiency provide valuable insights into the practical utility of these frameworks. Cross‐cultural and comparative studies are also needed to validate the applicability and adaptability of HCI frameworks in varied healthcare environments and user populations.

## Author Contributions

**Niloofar Mohammadzadeh:** conceptualization, supervision, project administration, methodology, data curation, validation, formal analysis, investigation, resources, writing – original draft. **Fatemeh Lotfi:** conceptualization, writing – original draft, methodology, data curation, validation, formal analysis, resources, investigation. **Hamed Samadpour:** conceptualization, writing – original draft, writing – review and editing, visualization, methodology, data curation, validation, formal analysis, resources, investigation. All authors reviewed and approved the final version of the manuscript.

## Ethics Statement

The authors have nothing to report.

## Consent

All authors consent to the publication of this manuscript in its final form. The manuscript has not been published previously, is not under consideration elsewhere, and has not been posted on a preprint server. The authors do not support preprinting of this manuscript and confirm that it will not be submitted to any preprint platform.

## Conflicts of Interest

The authors declare no conflicts of interest.

## Transparency Statement

The lead author Hamed Samadpour affirms that this manuscript is an honest, accurate, and transparent account of the study being reported; that no important aspects of the study have been omitted; and that any discrepancies from the study as planned (and, if relevant, registered) have been explained.

## Data Availability

All data supporting the findings of this study are available within the article. Additional materials or clarification are available from the corresponding author upon reasonable request. The corresponding author had full access to all data in the study and takes complete responsibility for the integrity of the data and the accuracy of the data analysis.
